# P-20. Associations of Comorbidities and Symptom Duration with Outcomes Among Patients with Staphylococcus aureus Bacteremia

**DOI:** 10.1093/ofid/ofaf695.251

**Published:** 2026-01-11

**Authors:** Felicia Ruffin, Vance G Fowler, Jeff Jabbour, Joshua T Thaden

**Affiliations:** Duke University Medical Center, Durham, NC; Duke University Medical Center, Durham, NC; Duke University Medical Center, Durham, NC; Duke University School of Medicine, Durham, NC

## Abstract

**Background:**

Chronic comorbid conditions represent significant risk for infection in patients with *Staphylococcus aureus* bacteremia (SAB) ^1^. How these conditions impact symptom duration (delays in diagnosis) and outcomes, i.e., high morbidity and mortality is poorly understood ^2^. Our objective was to investigate the associations between comorbidities, symptom duration, and mortality outcomes.

**Figure:**
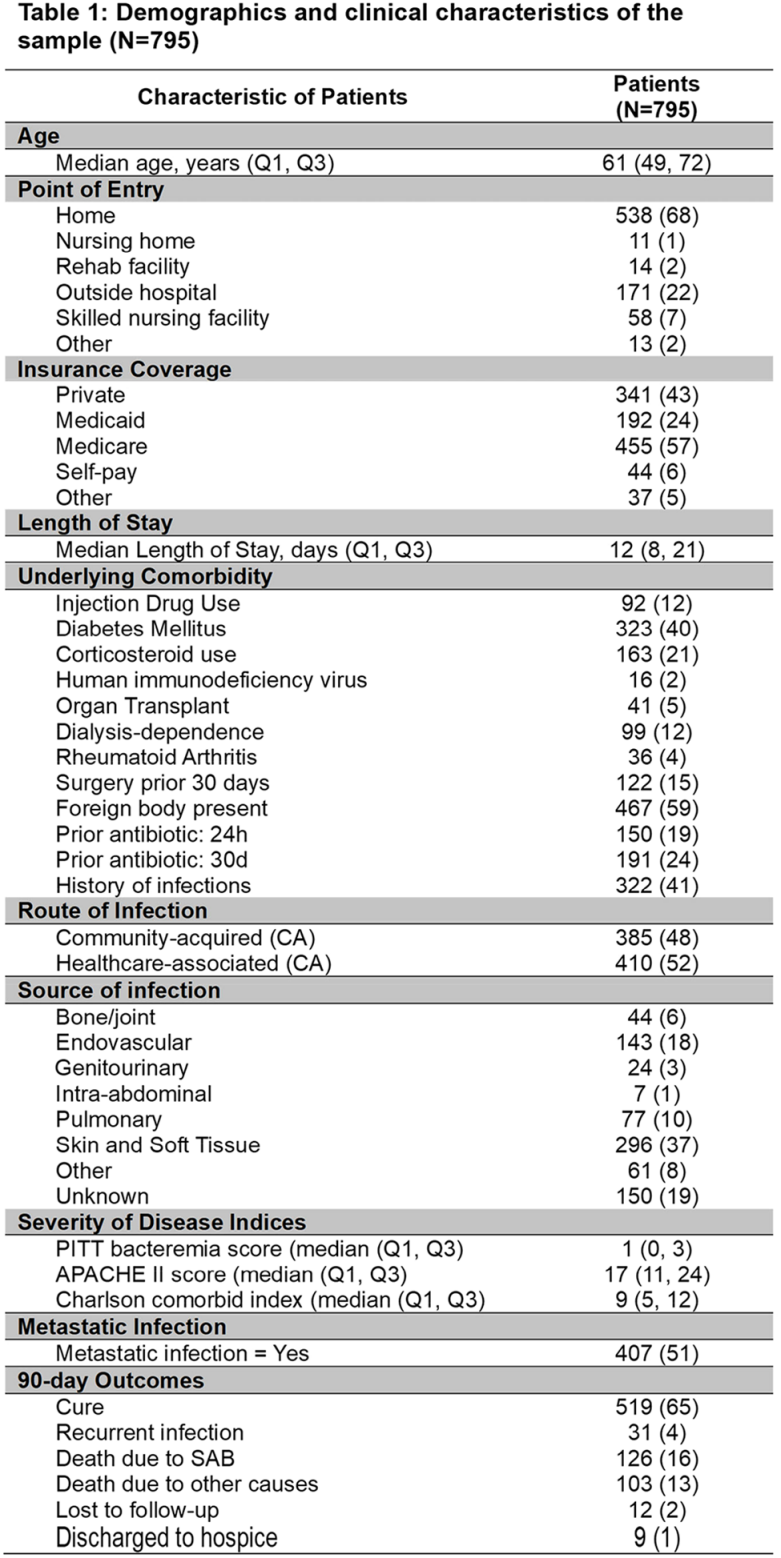

**Figure:**
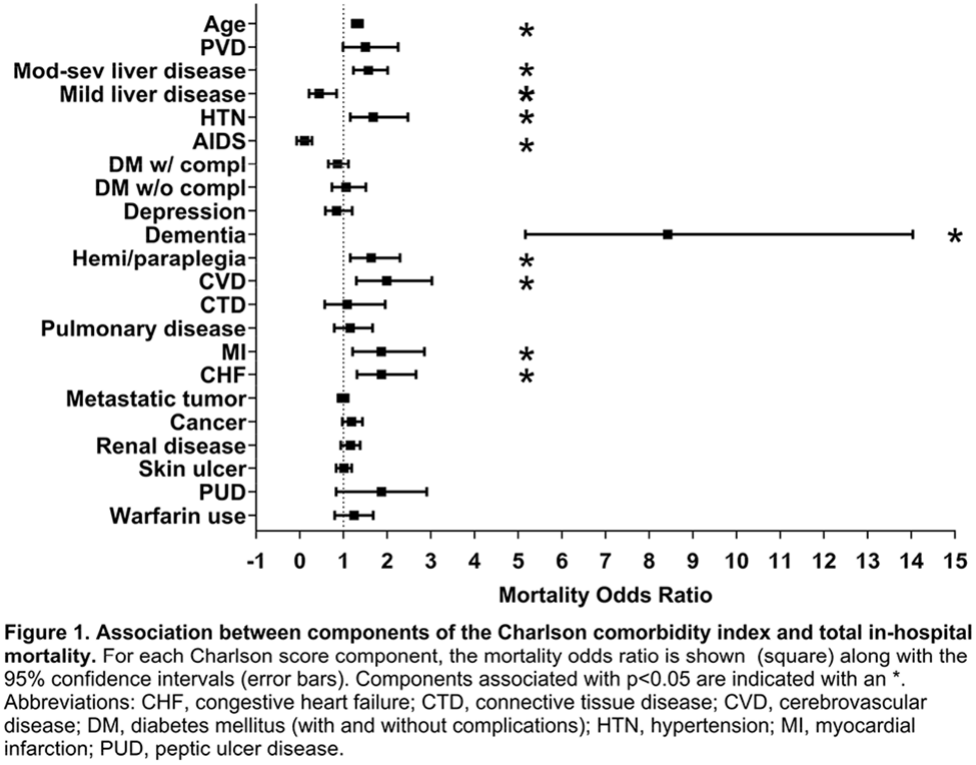

**Methods:**

This cohort study is a secondary analysis of data from patients who were prospectively enrolled in the Bloodstream Infections Registry at Duke University from July 2015 to August 2022. Participants included adults 18 years or older with a diagnosis of healthcare-associated and non-healthcare-associated community-acquired (HCA-CA & NHCA-CA) SAB. We used univariable and multivariable linear and logistic regression analyses to examine how comorbidities and the duration of symptoms are associated with the mortality.

**Figure:**
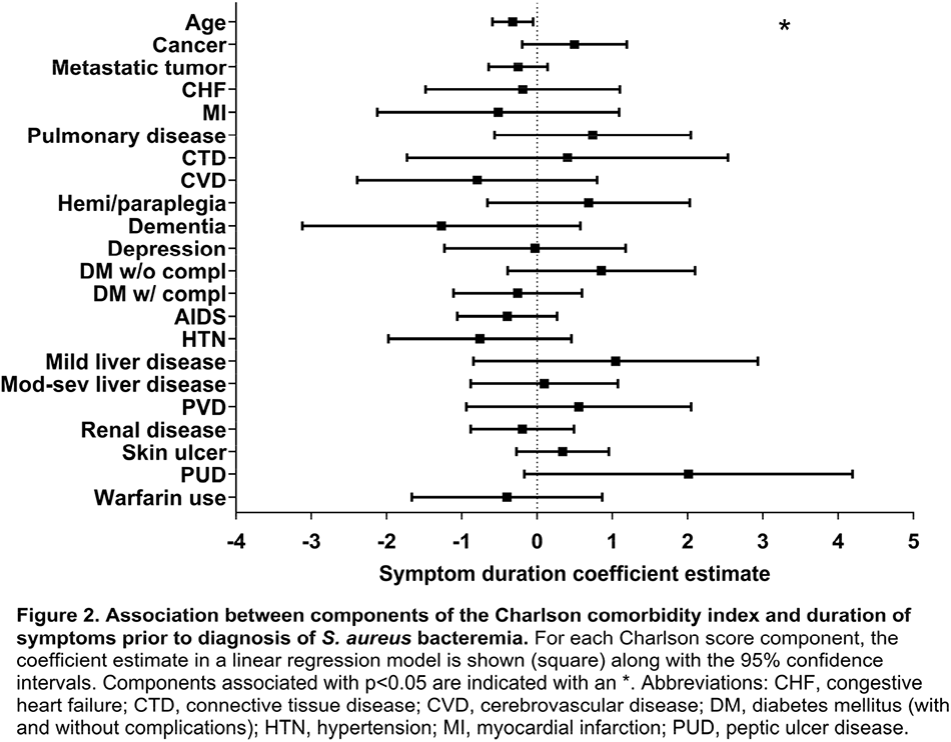

**Results:**

In total, 795 patients were included. Of these, 522 (66%) were White, and 500 (63%) were male. The median age was 61 (IQR, 49, 72). See Table 1 for complete descriptive statistics. The median number of days to diagnosis was 3 (IQR, 2, 6). Decreased symptom duration was statistically significantly associated with increased age (Linear regression estimate 0.32, 95% CI 0.59-0.05; p=0.02) (Figure 1). Multivariable regression analysis showed that patients with increased age ([OR] 1.31, CI 1.20-1.43; p=1.42), myocardial infarction ([OR] 1.86, CI 1.20-2.85; p=0.004), cerebrovascular accident ([OR] 1.99, CI 1.29-3.02; p=0.001), hemiplegia ([OR] 1.63, CI 1.15-2.29; p=0.004), hypertension ([OR] 1.67, CI 1.15-2.47; p=0.007), and severe liver disease ([OR] 1.56, CI 1.22-2.01; p < 0.00) were higher risk for mortality (Figure 2). The all-cause mortality rate was 29%, and patients with mild liver disease were less likely to die ([OR] 0.44, CI 0.21-0.84; p=0.01).

**Conclusion:**

In this cohort study of adults with SAB, comorbidities were associated with shorter symptom duration and an overall increased mortality risk. This study illustrates the importance of clinical management of comorbidities that increase patients’ for risk of infection and poor outcomes.

**Disclosures:**

Vance G. Fowler, MD, MHS, Affinergy, Janssen, Contrafect: Advisor/Consultant|AstraZeneca; EDE; Basilea: Grant/Research Support|Debiopharm, GSK; Affinium, Basilea,: Advisor/Consultant|Destiny, Amphliphi, Armata, Akagera: Advisor/Consultant|Merck; Contrafect; Karius; Janssen: Grant/Research Support|UpToDate: Royalties|Valanbio: Stock options

